# Supplementary material for: Hydrogel Surface-Modified Polyurethane Copolymer Film with Water Permeation Resistance and Biocompatibility for Implantable Biomedical Devices
Source: Micromachines (Basel). 2021 Apr 16;12(4):447. doi: 10.3390/mi12040447 (PMC8072913; doi:10.3390/mi12040447)
Supplement: Supplementary file 1 [file micromachines-12-00447-s001.zip › micromachines-1182165-supplementary.pdf]

Supplementary Materials

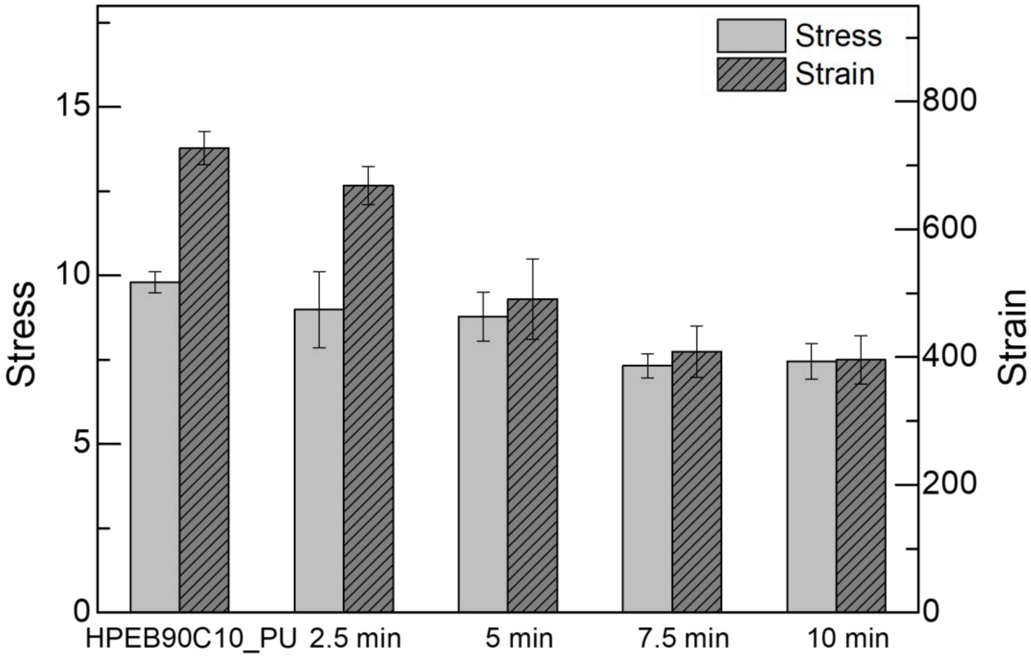

Figure S1. Tensile stress-strain value of HPEB90C10 with varying hexane treatment time.
